# Anlotinib combined with chemoradiotherapy for postoperative isolated lymph node recurrence of ESCC: a phase II study

**DOI:** 10.3389/fimmu.2026.1862131

**Published:** 2026-07-24

**Authors:** Jing Huang, Zhenlin Gu, Jinjing Xu, Changhua Yu, Weiguo Zhu, Yiyuan Zhang, Yong Gao

**Affiliations:** 1 Department of Radiation Oncology, The Affiliated Huai’an No. 1 People’s Hospital of Nanjing Medical University, Huai’an, Jiangsu, China; 2 Department of Vascular Surgery, The Affiliated Huai’an No. 1 People’s Hospital of Nanjing Medical University, Huai’an, Jiangsu, China; 3 Department of Basic Medicine, Jiangsu College of Nursing, Huai’an, Jiangsu, China; 4 Department of Nephrology, The Affiliated Huai’an No.1 People’s Hospital of Nanjing Medical University, Huai’an, Jiangsu, China; 5 Department of Oncology, The Affiliated Huai’an No. 1 People’s Hospital of Nanjing Medical University, Huai’an, Jiangsu, China

**Keywords:** anlotinib, chemoradiotherapy (CRT), esophageal squamous cell carcinoma (ESCC), isolated lymph node recurrence, radioresistance, transcriptomics

## Abstract

**Background:**

Postoperative lymph node recurrence in esophageal squamous cell carcinoma (ESCC) is associated with poor prognosis and lacks standardized treatment. Radioresistance and aberrant immune/inflammatory tumor microenvironment (TME) are major barriers limiting the efficacy of concurrent chemoradiotherapy (CRT). This study aimed to evaluate the efficacy, safety, and immune-related radiosensitising mechanisms of anlotinib combined with CRT for postoperative isolated lymph node recurrence of ESCC.

**Methods:**

In this single−arm phase II trial, 47 patients with postoperative isolated lymph node recurrence of ESCC received anlotinib plus concurrent CRT. Tumor response and safety were assessed per RECIST version 1.1 and NCI-CTCAE version 4.0. Peripheral blood immune indicators (CD4^+^/CD8^+^ ratio, TNF-α, IL-6) were dynamically monitored. Transcriptomic and pathway enrichment analyses were performed in radioresistant ESCC cells to explore immune−mediated radioresistance and anlotinib’s regulatory mechanisms.

**Results:**

The median progression−free survival (PFS) and overall survival (OS) were 20.2 and 30.7 months, respectively. The objective response rate (ORR) was 91.5% (12.8% complete response, 78.7% partial response), with a 100% disease control rate (DCR). The mean lymph node diameter reduction was 60.1 ± 18.0%. Treatment was well tolerated; Grade ≥ 3 treatment−related adverse events occurred in 42.6% of patients and were reversible without treatment interruption. Peripheral blood immune monitoring showed a significant increase in the CD4^+^/CD8^+^ ratio and marked decreases in TNF-α and IL-6 levels after treatment (all *P* < 0.001). Transcriptomic profiling revealed that acquired radioresistance in ESCC was accompanied by prominent enrichment of immune− and inflammation−related signaling pathways, including JAK-STAT, TNF, and cytosolic DNA−sensing pathways. Anlotinib may enhance radiosensitivity by normalizing tumor vasculature and remodeling the immunosuppressive TME, thereby alleviating immune−related radioresistance.

**Conclusions:**

Anlotinib combined with CRT achieves favorable efficacy and manageable toxicity in postoperative nodal recurrent ESCC. This regimen significantly reshapes the peripheral immune profile and overcomes radioresistance via dual modulation of tumor vasculature and immune-inflammatory pathways. It represents a promising salvage strategy worthy of validation in multicenter randomized controlled trials.

**Clinical trial registration:**

https://www.chictr.org.cn/, identifier ChiCTR1900023677.

## Introduction

Esophageal squamous cell carcinoma (ESCC) is a highly lethal malignancy, ranking eighth in global incidence and sixth in cancer-related mortality, with 604,100 new cases and 544,100 deaths in 2020 (1, 2). In China, ESCC accounts for over 90% of esophageal cancers, contributing to approximately 246,000 new diagnoses and 188,000 deaths annually (3, 4). High-incidence areas are concentrated in the “esophageal cancer belt,” including Henan, Hebei, Shanxi, Shandong, and Jiangsu provinces (5, 6). Northern Jiangsu-especially Huai’an, Xuzhou, and Lianyungang-exhibits age-standardized incidence rates exceeding 60–80 per 100,000, primarily attributed to nitrosamine exposure, poor oral hygiene, and limited screening (7). In these underdeveloped regions, delayed diagnosis and inadequate postoperative surveillance lead to high recurrence rates, reaching over 45% within two years (8, 9).

Isolated lymph node recurrence is a typical yet often unresectable relapse pattern in ESCC, posing substantial therapeutic challenges (10). Although concurrent chemoradiotherapy (CRT) is the standard salvage strategy, its efficacy is limited in this setting, with 2-year local control and overall survival (OS) rates ranging from 30% to 50%, primarily due to the intrinsic radioresistance of nodal lesions (11–15). Moreover, many postoperative patients exhibit reduced tolerance to systemic chemotherapy, highlighting the need for more effective and tolerable regimens (16–20).

Anlotinib is a novel oral tyrosine kinase inhibitor (TKI) that targets VEGFR, FGFR, PDGFR, and other angiogenic pathways, with demonstrated anti-tumor activity in thoracic malignancies (13–15). Beyond its antiangiogenic effects, preclinical studies suggest that anlotinib may enhance radiosensitivity by normalizing tumor vasculature and modulating the tumor microenvironment (TME) (21–23). Unlike mucosal or anastomotic relapses, isolated lymph node recurrence is associated with a lower risk of bleeding, providing a favorable setting for antiangiogenic therapy (18–20). Thus, this subgroup may benefit from the combined delivery of CRT and antiangiogenic agents, targeting a biologically aggressive yet anatomically manageable disease (22, 24). In this context, we conducted a prospective, single-arm, phase II trial to evaluate the efficacy and safety of anlotinib combined with CRT in patients with postoperative isolated lymph node recurrence of ESCC. In parallel, transcriptomic profiling of a radioresistant ESCC cell line (KYSE30-R) was performed to explore resistance mechanisms and investigate the radiosensitising potential of anlotinib. This dual clinical-translational approach aims to identify mechanistic correlates and inform biomarker-driven strategies in this high-risk population.

## Materials and methods

### Study population

**Figure 1:**
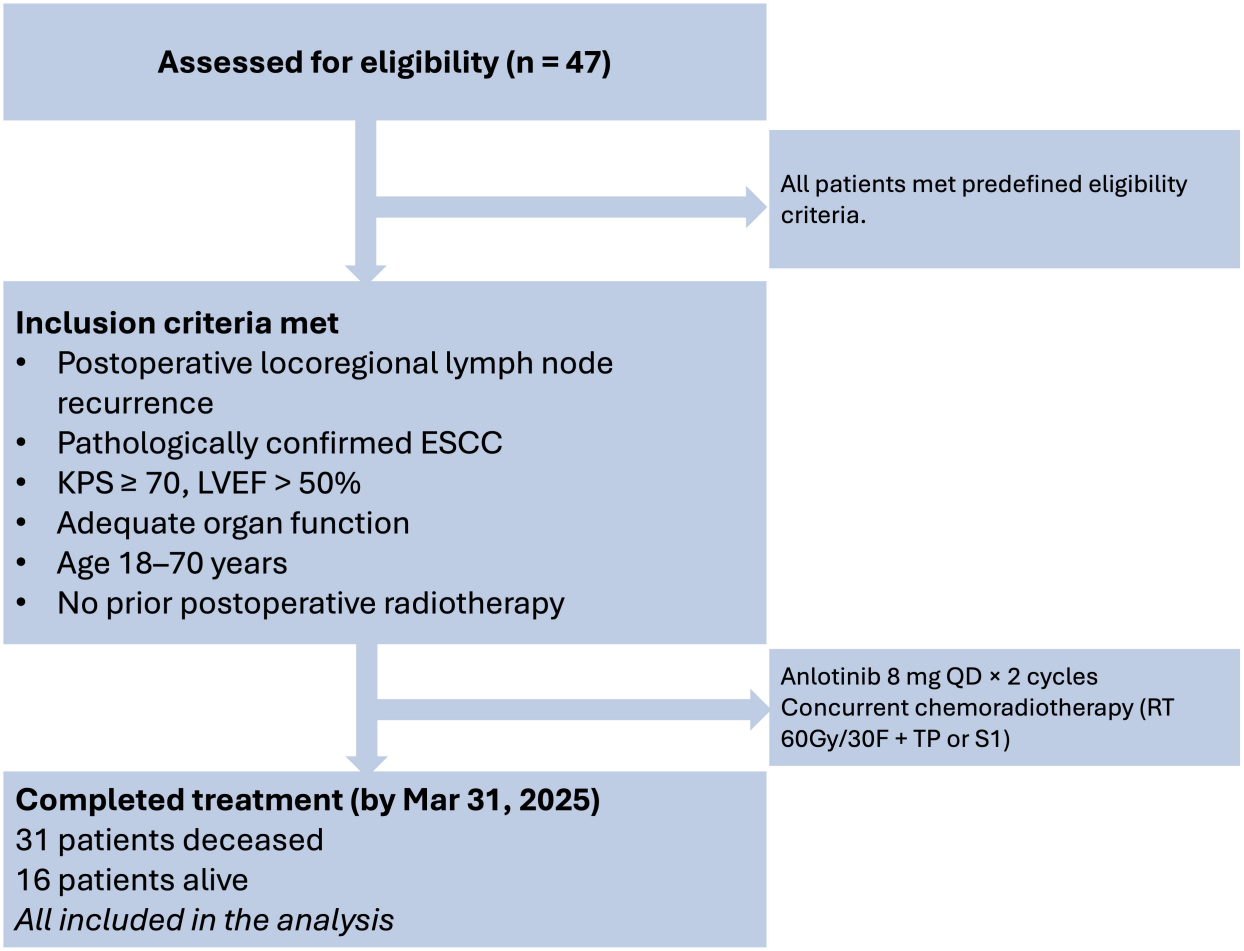
CONSORT-style flow diagram illustrating enrolment, eligibility, treatment, and analysis. All patients met the predefined inclusion criteria and received anlotinib-based concurrent CRT (60 Gy in 30 fractions with either TP or S-1 regimens) and were included in the final analysis.

This single-center, single-arm phase II trial was conducted at Huai’an First People’s Hospital, affiliated with Nanjing Medical University, starting in June 2019. The study was approved by the Institutional Review Board (Approval No. KY-P-2019-034-01), and written informed consent was obtained from all participants prior to their participation. The trial was registered with the Chinese Clinical Trial Registry (ChiCTR1900023677). Eligible patients were aged 18–70 years with pathologically confirmed ESCC and radiologically defined locoregional lymph node recurrence after curative esophagectomy (≤ 6 metastatic lymph nodes, no distant metastasis, no mucosal/anastomotic involvement, confirmed by chest/abdominal contrast-enhanced CT and esophageal barium swallow). Additional criteria included a Karnofsky Performance Status (KPS) ≥ 70, no prior postoperative radiotherapy, adequate hematologic, hepatic, renal, and cardiac function, and an estimated life expectancy of ≥ 12 weeks. The study adhered to the Declaration of Helsinki and Good Clinical Practice (ICH-GCP) guidelines. The protocol followed the SPIRIT (Standard Protocol Items: Recommendations for Interventional Trials) checklist, and the final report complied with the CONSORT (Consolidated Standards of Reporting Trials) guidelines. The enrollment process is illustrated in the CONSORT flow diagram (**Figure 1**).

### Treatments

Radiotherapy was delivered using intensity-modulated radiation therapy (IMRT) with 6–10 MV X-rays generated by an Elekta Infinity or Elekta Versa HD linear accelerator. Patients were immobilized in the supine position using a thermoplastic body mask, and contrast-enhanced CT simulation was performed with a 3-mm slice thickness from the skull base to the diaphragm. The gross tumor volume of involved lymph nodes (GTVnd) was defined as radiographically visible nodal lesions with a short-axis diameter > 1 cm on diagnostic CT, or nodes < 1 cm showing increased fluorodeoxyglucose uptake on Positron Emission Tomography/Computed Tomography (PET/CT), nodal fusion, or clustering. For reproducible radiological delineation, nodal fusion was defined as two or more adjacent lymph nodes with obliterated fat planes between them; a lymph node cluster was defined as ≥3 contiguous subcentimeter metastatic lymph nodes distributed within a single lymphatic drainage region without separating fatty tissue. The clinical target volume (CTV) encompassed the lymphatic drainage region containing the metastatic nodes, adjusted according to anatomic boundaries and patterns of spread. The planning target volume (PTV) was generated by expanding the CTV with a uniform 0.5 cm isotropic margin to account for setup uncertainty and respiratory motion. The prescribed radiation dose was 60 Gy in 30 fractions (2.0 Gy per fraction, once daily, 5 days per week). The prescription dose covered at least 95% of the PTV. Dose constraints for organs at risk (OARs) were defined per modern ICRU radiation oncology reporting standards as follows: spinal cord maximum point dose (D_max_) ≤ 45 Gy; lung V_20_ ≤ 25% and mean lung dose ≤ 15 Gy (V_20_ calculated based on total bilateral lung volume excluding gross tumor volume of metastatic lymph nodes [GTVnd]); heart V_40_ ≤ 30%; and stomach/esophagus (if overlapping with target volume) maximum dose to 1 cm^3^ tissue ≤ 55 Gy. In clinical practice, 50 Gy is the standard dose for definitive or postoperative adjuvant radiotherapy in primary ESCC. However, the patients in this study suffered from postoperative locoregional recurrence, and the treatment administered was salvage radiotherapy. For recurrent lesions, the local control rate of 50 Gy is relatively insufficient. Clinical consensus and multiple retrospective studies worldwide have consistently demonstrated that a dose escalation to approximately 60 Gy can significantly improve local tumor control and achieve better radical therapeutic effects for salvage radiotherapy. Dose distribution and plan optimization were evaluated using dose-volume histograms (DVHs). Image-guided verification (cone-beam CT) was performed before each treatment session to ensure accurate setup.

Concurrent chemotherapy commenced on the first day of radiotherapy. Depending on performance status and comorbidities, patients received either weekly paclitaxel (80 mg/m^2^) plus cisplatin (25 mg/m^2^), or S-1 monotherapy (80 mg/m^2^/day) administered orally in two divided doses within 30 minutes after breakfast and dinner, at the treating physician’s discretion (25–28). Anlotinib was started at a dose of 8 mg once daily, administered orally on an empty stomach. The schedule consisted of two weeks on treatment followed by one week off, with one cycle defined as 3 weeks, for a total of 2 cycles (6 weeks) of planned administration, or until radiographic progression or intolerable toxicity. If the patient had no progressive disease and tolerable toxicity after 2 cycles, anlotinib was administered at a reduced dose of 6 mg once daily (2 weeks on/1 week off) for maintenance treatment until disease progression. Treatment continued until the completion of the planned course or earlier discontinuation due to disease progression or severe adverse events (**Supplementary Figure 1**).

### Patient assessment

Baseline evaluations included a comprehensive physical examination, KPS assessment, laboratory tests (complete blood count, serum chemistry, coagulation profile, and urinalysis), electrocardiogram (ECG), esophageal barium swallow, and chest/abdomen CT scans. Baseline peripheral blood samples were collected to detect immune-related indicators (lymphocyte subpopulations, serum cytokines including TNF-α, IL-6). During CRT, patients were assessed weekly for clinical symptoms, hematological parameters, and blood pressure. Comprehensive safety assessments were conducted on Days 14, 28, and 42, which included biochemical markers, ECGs, and pulmonary function tests. Radiologic and clinical evaluations of tumor response were conducted on Days 70 ± 7 and 126 ± 7, and every 56 ± 7 days thereafter during the first year. Tumor response was assessed according to the Response Evaluation Criteria in Solid Tumors (RECIST) version 1.1. All adverse events (AEs) were documented and graded based on the National Cancer Institute Common Terminology Criteria for Adverse Events (NCI-CTCAE) version 4.0.

### Outcomes

The primary endpoint was progression-free survival (PFS), defined as the time from study enrolment to the first occurrence of disease progression or death from any cause, whichever occurred first. OS was a key secondary endpoint, calculated from the date of study entry to the date of death from any cause. Additional secondary endpoints included the objective response rate (ORR) and disease control rate (DCR), assessed according to the RECIST, version 1.1. Subgroup analyses were performed based on the number of metastatic lymph nodes (≤ 3 vs. 4-6), the maximum diameter of lymph nodes (≥ 4 cm vs. < 4 cm). Treatment responses were evaluated through serial imaging and clinical assessments. Safety outcomes included the incidence and severity of treatment-related adverse events (TRAEs) and serious adverse events (SAEs), graded according to the NCI-CTCAE version 4.0.

### Establishment of radioresistant KYSE30-R cells

Radioresistant KYSE30-R cells were generated using a stepwise fractionated irradiation protocol based on previously established methods for inducing radioresistance in ESCC cell lines. Parental KYSE30 cells (from the Chinese Type Culture Collection, Shanghai, China) were seeded in 25 cm^2^ flasks and irradiated with a high-energy linear accelerator (Elekta Versa HD, Elekta AB, Stockholm, Sweden) equipped with 1.5 mm tissue-equivalent compensation at a dose rate of 100 cGy/min. The irradiation schedule consisted of 1 Gy×3, 2 Gy×3, and 4 Gy×3 fractions (9 fractions in total), delivered twice weekly over 5 weeks (29). Following irradiation, cells were maintained under standard culture conditions (RPMI-1640 supplemented with 10% fetal bovine serum [FBS, AusGeneX, Australia] and 1% penicillin-streptomycin [Gibco, USA]) and passaged without further irradiation for at least one month before subsequent experiments.

All cell lines used in this study were authenticated by an experienced pathologist and cell biology expert before the experiment, and the authentication results confirmed that the cell lines were consistent with the target cell type without misidentification.

Before the experiment, mycoplasma detection was performed on all used cell lines using the mycoplasma detection kit (Cat. No.: HY-K0552), and the detection results showed that all cell lines were free from mycoplasma contamination.

### RNA sequencing and bioinformatic analysis

Total RNA was extracted from radioresistant (Ra) and parental (NC) KYSE30 cells using TRIzol reagent (Invitrogen, USA). RNA quality was assessed with NanoDrop 2000, Qubit 2.0, and Agilent 2100 Bioanalyzer. Samples with an OD260/280 ratio between 1.8 and 2.2, an OD260/230 ratio ≥ 2.0, and an RNA integrity number (RIN) ≥ 7.0 were included for sequencing. RNA-seq libraries were prepared using the NEBNext Ultra RNA Library Prep Kit with poly(A) selection and sequenced on the Illumina NovaSeq 6000 platform (paired-end, 150 bp). Raw reads were filtered using fastp (version 0.20.0) and then aligned to the GRCh38 human reference genome with HISAT2 (version 2.1.0) (30, 31). Transcript assembly and quantification were performed with StringTie (v1.3.4d) (32). Differentially expressed genes (DEGs) were identified using DESeq2 (fold-change > 2; FDR < 0.05) (33). Functional enrichment analyses were performed using clusterProfiler for Gene Ontology (GO) and Kyoto Encyclopedia of Genes and Genomes (KEGG) pathways. Gene Set Enrichment Analysis (GSEA) was conducted with fgsea based on MSigDB gene sets. Transcription factor (TF) regulatory networks were inferred using AnimalTFDB3 and visualized with Cytoscape (v3.8.0). All analyses were conducted in R (v4.0.2) (34–36).

### Statistical analysis

#### Sample size calculation

This was a single−arm, open−label, Phase II clinical trial employing a Simon two-stage design. The primary endpoint was progression-free survival (PFS). Assuming PFS followed an exponential distribution, the historical control median PFS was set at 11.5 months, and the expected median PFS for the anlotinib-based regimen was 20.2 months (hazard ratio [HR] ≈ 0.57). With a one−sided α of 0.05, a power of 1 - β = 0.80, and a 20% dropout rate, the total required sample size was calculated to be at least 25 patients, which was consistent with the actual enrolled sample size.

#### Data processing and analysis

Statistical analyses were performed using R (v4.0.2) and GraphPad Prism (v10.3.1). PFS and OS were estimated with the Kaplan-Meier method and compared using the log-rank test. Univariate and multivariate Cox proportional hazards models were applied to identify independent prognostic factors. Categorical variables were compared using the Chi-square test or Fisher’s exact test, while continuous variables were compared using the Student’s t-test or Mann-Whitney U test, as appropriate. RNA-seq differential expression analysis was conducted using DESeq2, with an *FDR* < 0.05 considered significant. A two-sided *P* < 0.05 was deemed statistically significant for all other comparisons.

## Results

### Patient characteristics

A total of 47 patients with postoperative locoregional lymph node recurrence of ESCC were included. The median age was 64.8 ± 5.9 years, and 77% were male (36/47). Most patients (91.5%) had a KPS ≥ 80, while the remaining 8.5% had a KPS of 70. The most common comorbidities were hypertension (11%), diabetes mellitus (6.4%), and coronary artery disease (2.1%) (**Table 1**). All patients had previously undergone curative esophagectomy. At recurrence, the mean diameter of the involved lymph nodes was 4.6 ± 0.9 cm, which decreased to 1.8 ± 0.9 cm following treatment. This corresponded to an average absolute reduction of 2.76 ± 0.98 cm and a mean shrinkage rate of 60.1 ± 18.0 %. The recurrent lymph node characteristics were: number (≤ 3: 63.8%, 4-6: 36.2%); maximum diameter (≥ 4 cm: 59.6%, < 4 cm: 40.4%). Two concurrent CRT regimens were administered: S-1 monotherapy in 21% of patients and paclitaxel plus cisplatin (TP) in 79%. According to RECIST version 1.1, the initial tumor response included complete response (CR) in 13% (6/47), partial response (PR) in 79% (37/47), and stable disease (SD) in 8.5% (4/47). Baseline peripheral blood immune indicators showed that the CD4^+^/CD8^+^ ratio was 1.2 ± 0.4, and the serum levels of TNF-α and IL-6 were 15.6 ± 4.2 pg/mL and 22.3 ± 5.8 pg/mL, respectively.

**Table 1:** Baseline characteristics of the study population.

Variable	N = 47
Gender	
Female	11 (23%)
Male	36 (77%)
Age (years)	64.8 ± 5.9
KPS score	
70	4 (8.5%)
80	30 (64%)
90	13 (28%)
Comorbidities	
Hypertension	5 (11%)
Diabetes	3 (6.4%)
Coronary heart disease	1 (2.1%)
Tumour characteristics	
Baseline size (cm)	4.6 ± 0.9
Post-RT size (cm)	1.8 ± 0.9
Lesion shrinkage (cm)	2.76 ± 0.98
Lesion shrinkage rate (%)	60.1 ± 18.0 %
Prior therapy	Surgery: 47 (100%)
Characteristics of regional lymph node mecurrence	
Number Maximum diameter (cm) Concurrent chemoradiotherapy	≤3: 30 (63.8%), 4-6: 17 (36.2%) <4 cm: 19 (40.4%), ≥4 cm: 28 (59.6%) 47 (100%)
Radiotherapy	60Gy/30F
Chemotherapy	S-1: 10 (21%), TP: 37(79%)
Tumour response (RECIST)	
CR	6 (12.8%)
PR	37 (78.7%)
SD	4 (8.5%)

Unless otherwise indicated, data are presented as n (%) or mean ± standard deviation (SD). N = 47.

Demographic and clinical features of 47 patients with postoperative locoregional lymph node recurrence of ESCC, including age, sex, comorbidities, KPS, treatment details, and tumor response by RECIST version 1.1.

### Survival outcomes and subgroup analysis

**Figure 2:**
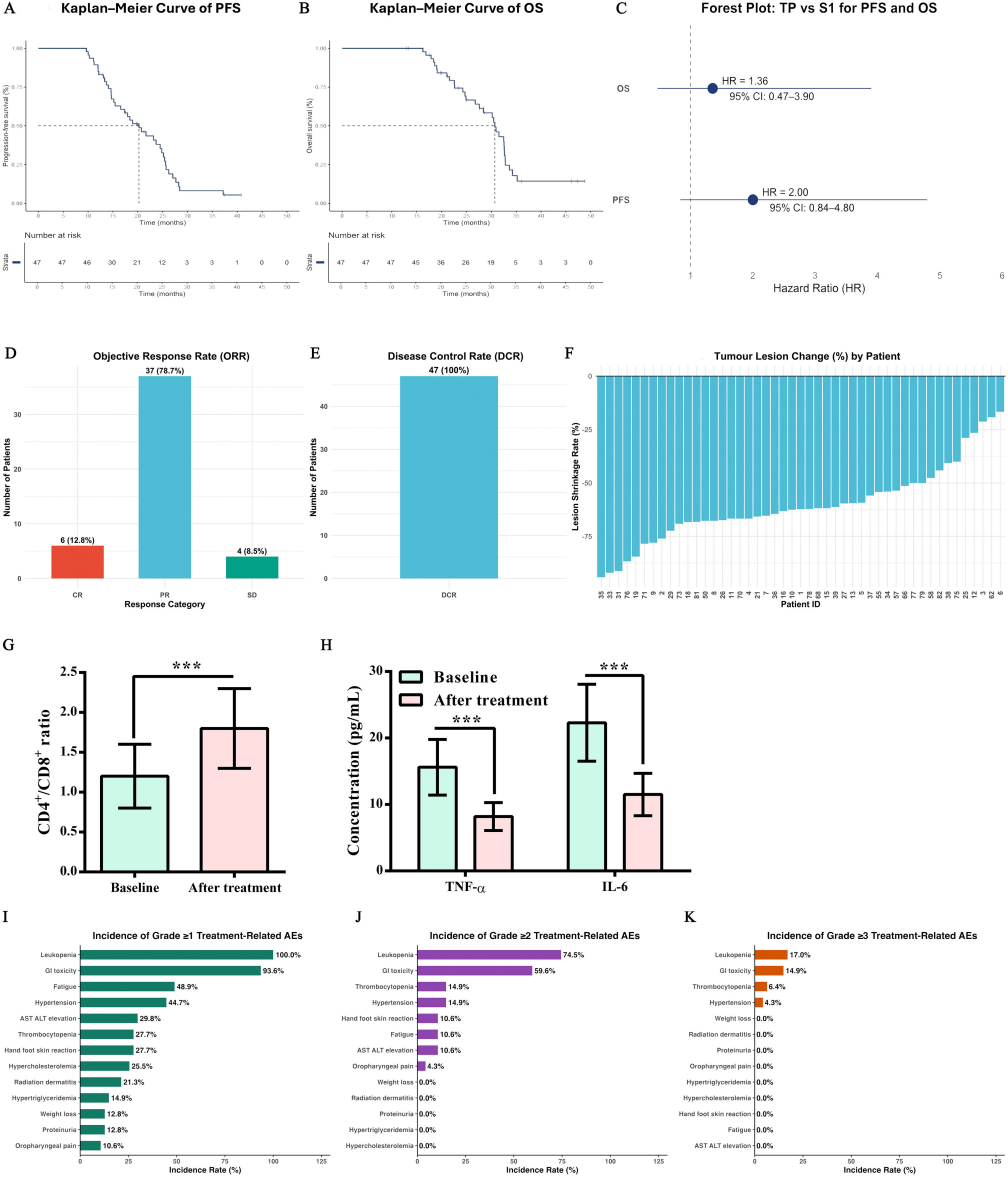
Efficacy, safety and immune biomarkers of anlotinib-based concurrent CRT for recurrent ESCC with isolated postoperative lymph node metastasis. Kaplan-Meier curves of **(A)** PFS and **(B)** OS in 47 patients with postoperative isolated lymph node recurrence of ESCC treated with anlotinib-based concurrent CRT. **(C)** Forest plot comparing TP versus S-1 regimens for PFS and OS; no statistically significant differences were observed. **(D-F)** Tumor response to anlotinib-based concurrent CRT in 47 patients with postoperative isolated lymph node recurrence of ESCC: **(D)** ORR: complete response (CR), partial response (PR), and stable disease (SD). **(E)** DCR, defined as CR + PR + SD. **(F)** Waterfall plot showing individual lesion shrinkage; no patient exhibited progressive disease (≥ 20% increase in size). **(G)** The CD4^+^/CD8^+^ T-cell ratio was measured at baseline and following treatment. **(H)** The concentrations of tumor necrosis factor-alpha (TNF-α) and interleukin-6 (IL-6) were assessed. **(I-K)** TRAEs in 47 patients with postoperative isolated lymph node recurrence of ESCC receiving anlotinib-based concurrent CRT: **(I)** Grade ≥ 1 TRAEs. **(J)** Grade ≥ 2 TRAEs. **(K)** Grade ≥ 3 TRAEs. Leukopenia and gastrointestinal toxicity were the most frequent. No Grade 4 events or treatment-related deaths occurred. Percentages indicate the proportion of the total cohort. ***: p < 0.001.

All 47 patients received anlotinib (8 mg once daily, 2 weeks on/1 week off for two cycles) concurrently with radiotherapy (60 Gy in 30 fractions) and either S-1 or TP chemotherapy. At the data cut-off (31 March 2025), 31 patients had died, and 16 were still alive. The median follow-up time, estimated using the reverse Kaplan-Meier method, was 36.1 months. The median PFS was 20.2 months (95% CI: 16.6–25.2), and the median OS was 30.7 months (95% CI: 27.6–32.8) (**Figures 2A, B**). These results indicate promising long-term outcomes in this high-risk postoperative population. To assess the prognostic impact of the chemotherapy regimen, a subgroup analysis was performed comparing the TP and S-1 groups. Univariate Cox regression revealed no significant difference in PFS (HR = 2.00; 95% CI: 0.84–4.80; *P* = 0.12) or OS (HR = 1.36; 95% CI: 0.47–3.90; *P* = 0.57) between the two groups (**Figure 2C**; **Supplementary Table S1**). Subgroup analyses based on lymph node characteristics showed that the median PFS and OS were not significantly different between the subgroups of Number of recurrent lymph nodes (≤ 3 vs. 4–6), Maximum diameter of recurrent lymph nodes (≥ 4 cm vs. < 4 cm) (all *P* > 0.05, **Supplementary Figure 2**).

### Tumor response evaluation

Objective tumor response was assessed in all 47 patients. As shown in **Table 2** and **Figure 2D**, the objective response rate (ORR) was 91.5%, comprising 6 complete responses (12.8%) and 37 partial responses (78.7%). The remaining 4 patients (8.5%) achieved stable disease (SD), resulting in a disease control rate (DCR) of 100% (**Figure 2E**). Quantitative analysis demonstrated a mean absolute reduction in lymph node diameter of 2.8 ± 1.3 cm, corresponding to a mean relative shrinkage rate of 60.1 ± 18.0%. The median shrinkage rate was 62.2%, with a range of 16.7% to 94.1%, indicating consistent and substantial tumor regression. No cases of progressive disease (PD) were observed during the assessment period. A waterfall plot (**Figure 2F**) illustrated the distribution of individual responses. After treatment, the peripheral blood CD4^+^/CD8^+^ ratio increased to 1.8 ± 0.5, and the serum levels of TNF-α and IL-6 decreased to 8.2 ± 2.1 pg/mL and 11.5 ± 3.2 pg/mL, respectively (all *P* < 0.001 vs. baseline, **Figures 2G, H**; **Table 3**).

**Table 2:** Tumor response according to RECIST version 1.1.

Tumour response	n (%)
Complete response (CR)	6 (12.8%)
Partial response (PR)	37 (78.7%)
Stable disease (SD)	4 (8.5%)
Objective response rate (ORR) (CR + PR)	43 (91.5%)
Disease control rate (DCR) (CR + PR + SD)	47 (100%)

Data are presented as n (%), unless otherwise specified.

RECIST, Response Evaluation Criteria in Solid Tumours.

Best overall response, including CR, PR, SD, ORR, and DCR, in 47 patients with postoperative locoregional lymph node recurrence of ESCC.

**Table 3:** Comparison of immune parameters and inflammatory cytokine levels before and after the intervention.

Indicators	Baseline	After treatment	P value
CD4^+^/CD8^+^ ratio	1.2 ± 0.4	1.8 ± 0.5	<0.001
TNF-α (pg/mL)	15.6 ± 4.2	8.2 ± 2.1	<0.001
IL-6 (pg/mL)	22.3 ± 5.8	11.5 ± 3.2	<0.001

TNF-α, Tumor Necrosis Factor−α; IL-6, Interleukin−6.

### Treatment-related adverse events

All 47 patients were evaluable for treatment-related adverse events (TRAEs), as summarized in **Table 4** and **Figures 2I–K**. The most common any-grade TRAEs were leukopenia (100%), gastrointestinal (GI) toxicity (93.6%), and fatigue (48.9%). Grade ≥ 1 events occurred in all patients (**Figure 2I**), while Grade ≥ 2 events were observed in 74.5%, most frequently leukopenia (74.5%) and GI toxicity (59.6%) (**Figure 2J**). Grade ≥ 3 TRAEs were reported in 20 patients (42.6%), primarily leukopenia (17.0%) and GI toxicity (14.9%), followed by thrombocytopenia (6.4%) and hypertension (4.3%) (**Figure 2K**). All Grade ≥ 3 TRAEs were effectively controlled after symptomatic treatment, and no patients required treatment discontinuation or dose reduction due to TRAEs. No Grade 4 events or treatment-related deaths were observed. Overall, the toxicity profile was manageable and consistent with the expected safety spectrum of anlotinib-based CRT, which was administered in 79% (37/47) of patients. Treatment adherence was high, with no dose-limiting toxicities or treatment discontinuations due to adverse events.

**Table 4:** TRAEs with anlotinib-based concurrent CRT.

Toxicities	Any grade (%)	G1 (%)	G2 (%)	G3 (%)	G4 (%)
Hypertriglyceridemia	7 (14.9%)	7 (14.9%)	0 (0.0%)	0 (0.0%)	0 (0.0%)
Proteinuria	6 (12.8%)	6 (12.8%)	0 (0.0%)	0 (0.0%)	0 (0.0%)
Weight loss	6 (12.8%)	6 (12.8%)	0 (0.0%)	0 (0.0%)	0 (0.0%)
Oropharyngeal pain	5 (10.6%)	3 (6.4%)	2 (4.3%)	0 (0.0%)	0 (0.0%)
Leukopenia	47 (100.0%)	12 (25.5%)	27 (57.4%)	8 (17.0%)	0 (0.0%)
GI toxicity	44 (93.6%)	16 (34.0%)	21 (44.7%)	7 (14.9%)	0 (0.0%)
Fatigue	23 (48.9%)	18 (38.3%)	5 (10.6%)	0 (0.0%)	0 (0.0%)
Hypertension	21 (44.7%)	14 (29.8%)	5 (10.6%)	2 (4.3%)	0 (0.0%)
AST ALT elevation	14 (29.8%)	9 (19.1%)	5 (10.6%)	0 (0.0%)	0 (0.0%)
Hand foot skin reaction	13 (27.7%)	8 (17.0%)	5 (10.6%)	0 (0.0%)	0 (0.0%)
Thrombocytopenia	13 (27.7%)	6 (12.8%)	4 (8.5%)	3 (6.4%)	0 (0.0%)
Hypercholesterolemia	12 (25.5%)	12 (25.5%)	0 (0.0%)	0 (0.0%)	0 (0.0%)

Adverse events were graded according to CTCAE version 5.0.

GI, gastrointestinal; AST, aspartate transaminase; ALT, alanine transaminase.

Incidence of any-grade and grade-specific (G1-G4) events in patients receiving anlotinib plus concurrent CRT. Data are shown as n (%). No G4 events occurred.

### Transcriptomic profiling of radioresistant ESCC cells

**Figure 3:**
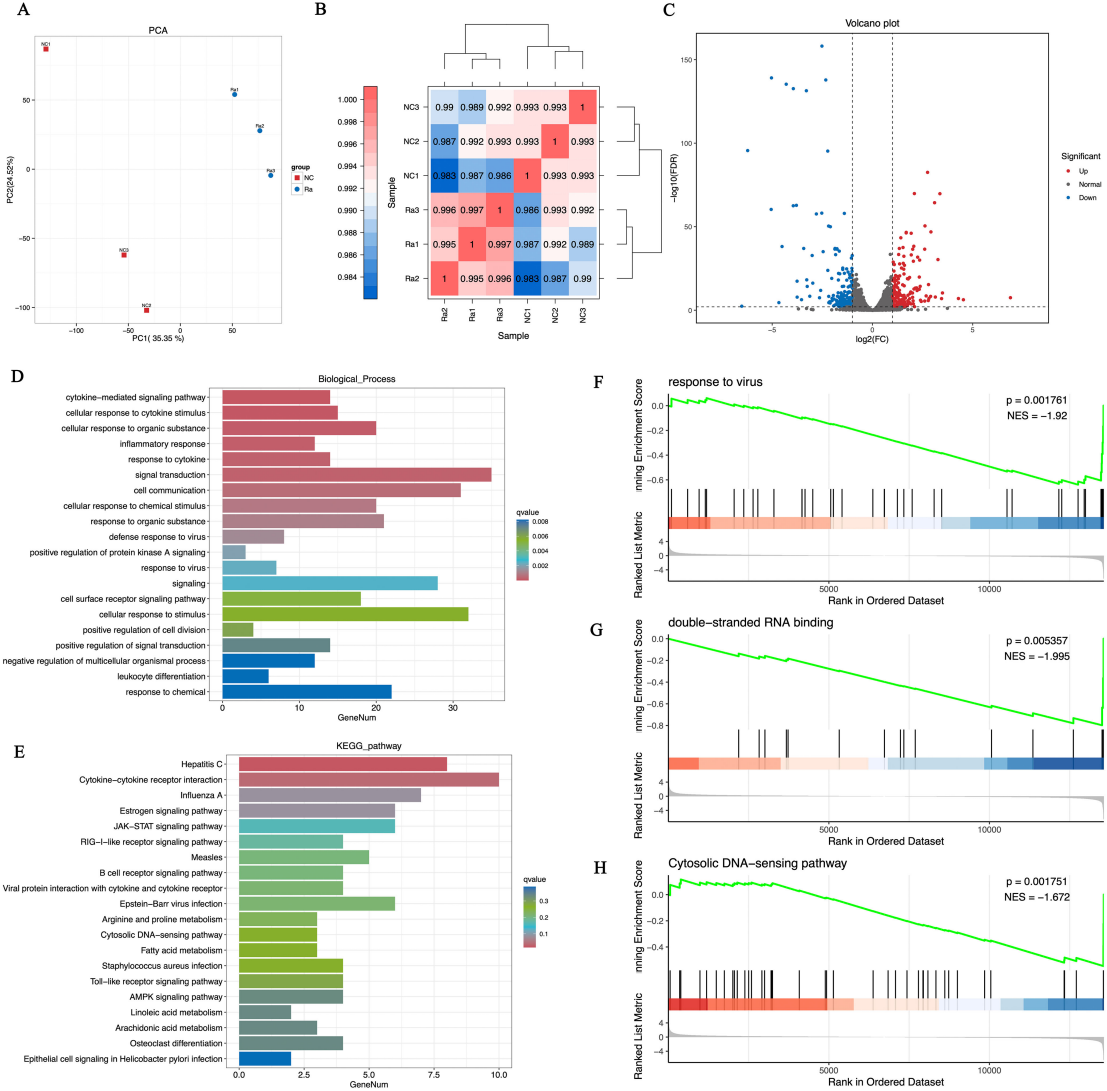
Transcriptomic profiling and functional enrichment of radioresistant KYSE30 cells. **(A)** PCA showing distinct separation between parental (NC) and radioresistant (Ra) cells. **(B)** Pairwise correlation heatmap demonstrating high intra-group and low inter-group similarity. **(C)** Volcano plot of differentially expressed genes (|log_2_FC| > 1, *FDR* < 0.05). Red and blue indicate significant up- and downregulation in Ra cells; grey indicates non-significant changes. **(D)** GO biological process enrichment highlighting immune-related terms such as cytokine-mediated signaling, inflammatory response, and cellular response to stimulus (*q* < 0.01). **(E)** KEGG pathway enrichment including cytokine–cytokine receptor interaction, JAK–STAT signaling, TNF signaling, and NOD-like receptor signaling (*q* < 0.01). **(F–H)** GSEA showing suppression of innate immune and nucleic acid sensing pathways in Ra cells: response to virus (NES = –1.92, *P* = 0.0018), double-stranded RNA binding (NES = –2.00, *P* = 0.0054), and cytosolic DNA-sensing (NES = –1.67, *P* = 0.0018).

To investigate the molecular basis of clinical radioresistance in ESCC, KYSE30-R was generated through repeated fractionated irradiation of the parental cells. Transcriptomic profiling was performed on both Ra and non-irradiated control (NC) cells using RNA-seq. Quality control verified consistent global expression across biological replicates. The density distribution of log_10_(FPKM) values showed comparable transcriptomic distributions between the groups, supporting the validity of downstream analyses (**Supplementary Figure 3**). Principal component analysis (PCA) revealed a clear separation between NC and Ra samples, indicating distinct transcriptomic signatures (**Figure 3A**). Correlation heatmaps further confirmed intra-group homogeneity and inter-group divergence (**Figure 3B**). Differential expression analysis identified 354 significantly altered genes (*FDR* < 0.05), including 162 upregulated and 192 downregulated transcripts in Ra cells (**Figure 3C**; **Supplementary Figure 3**). A heatmap with hierarchical clustering demonstrated consistent DEGs-based grouping by radiation sensitivity status (**Supplementary Figure 3**). The complete list of DEGs is provided in **Supplementary Table 2**, and the raw count matrix in **Supplementary Table 5**. Overall, these findings reveal extensive transcriptomic reprogramming associated with acquired radioresistance in ESCC, providing a robust foundation for subsequent pathway analysis.

### Mechanistic insights from DEGs and pathway analyses

To clarify the biological mechanisms behind radioresistance in ESCC, functional enrichment analyses were performed on DEGs identified between Ra and NC KYSE30 cells. Gene Ontology (GO) and KEGG analyses revealed significant enrichment in immune-related processes, including cytokine-mediated signaling, the JAK-STAT cascade, TNF signaling, and NOD-like receptor pathways (**Figures 3D–E**; **Supplementary Table 4**). Gene Set Enrichment Analysis (GSEA) further confirmed suppression of core innate immune pathways in Ra cells. Significantly enriched gene sets included defense response to virus (NES = –1.92, *P* = 0.0018), double-stranded RNA binding (NES = –2.00, *P* = 0.0054), and cytosolic DNA-sensing pathways (NES = –1.67, *P* = 0.0018) (**Figures 3F–H**; **Supplementary Figure 4**). Detailed GSEA outputs for GO and KEGG gene sets are in **Supplementary Table 3** and S3B, respectively. To explore upstream regulatory mechanisms, TF network analysis was performed. GO enrichment of DEGs and heatmap visualization of TF influence scores identified key regulators potentially driving the observed transcriptomic reprogramming (**Supplementary Figure 5A**). Overall, these findings suggest that the suppression of cytosolic DNA sensing and other innate immune pathways in tumor cells, combined with the activation of inflammatory signaling axes including JAK-STAT and TNF pathways, jointly shape an immunosuppressive TME and drive the development of radioresistance in ESCC. Immune-related pathways and regulatory elements therefore play a crucial role in radioresistance and may represent potential therapeutic targets.

## Discussion

This prospective Phase II study indicates that anlotinib combined with concurrent chemoradiotherapy (CRT) achieves promising clinical efficacy and a manageable safety profile in esophageal squamous cell carcinoma (ESCC) patients with postoperative lymph node recurrence. The median progression-free survival (PFS) was 20.2 months and median overall survival (OS) was 30.7 months. Compared with previously reported salvage CRT regimens, our results appear to show a potential survival benefit in this patient population, for whom historical median PFS ranges from 9 to 14 months and median OS from 13 to 18 months (10, 37–40). Collectively, these results may help fill the unmet medical need for this high-risk subgroup with regional recurrent disease.

A significant strength of this study lies in the deliberate selection of patients with isolated lymph nodal recurrence-excluding mucosal and anastomotic lesions that pose a higher bleeding risk and are less responsive to antiangiogenic therapy. This approach allowed for a focused assessment of anlotinib in a biologically aggressive yet anatomically safer context. Given the inherent radioresistance of nodal metastases, enhancing the sensitivity of CRT is essential (41). Anlotinib, a multi-targeted tyrosine kinase inhibitor (TKI), blocks angiogenic pathways (VEGFR, FGFR, PDGFR) and exhibits radiosensitising properties by normalizing vasculature and modulating the tumor microenvironment (TME) (35, 36).

To investigate its mechanistic relevance, we conducted transcriptomic profiling on a KYSE30-R model. RNA sequencing and pathway enrichment analyses revealed the upregulation of immune and inflammatory signaling, particularly in the JAK-STAT and TNF pathways. Notably, the cytosolic DNA-sensing and other core innate immune pathways were significantly suppressed in radioresistant cells. The coexistence of activated chronic inflammatory pathways and inhibited innate immune sensing pathways collectively induces a chronic inflammatory state and forms an immunosuppressive TME, which is the key driver of radioresistance. These results indicate a state of chronic inflammation within an immunosuppressive TME that may contribute to radioresistance. Notably, treatment with anlotinib partially reduced VEGF-related and immunosuppressive transcript signatures, suggesting a potential rebalancing of vascular and immune functions. As outlined in **Figures 3D–H**, we propose a model in which anlotinib increases radiosensitivity by dual regulation of tumor vasculature and immune signaling: it reverses the inhibition of innate immune sensing pathways and mitigates excessive activation of inflammatory pathways, relieving the immunosuppressive state of TME and restoring anti-tumor immune surveillance, thereby enhancing CRT response.

These insights align with reports from other tumor types, where antiangiogenic agents enhance radiotherapy by improving perfusion, decreasing hypoxia, and modulating the immune environment (42). Our combination of clinical efficacy with transcriptomic analysis supports the idea that anlotinib not only acts as an antiangiogenic agent but also reduces immune-related radioresistance. Although the application of anlotinib in ESCC has been examined before, our study is among the first to merge prospective clinical data with transcriptomic profiling of radioresistant cells, emphasizing immune pathways that may contribute to therapeutic resistance. These findings offer a mechanistic basis for future research into anlotinib-based radiosensitisation strategies targeting the tumor microenvironment. However, the KYSE30-R radioresistant model used in this study is a simplified *in vitro* monolayer tumor cell system. The transcriptomic alterations, including the upregulation of JAK-STAT, TNF, and cytosolic DNA-sensing pathways, merely represent the intrinsic adaptive survival machinery of tumor cells under radiation stress. The direct causal link between these changes and the pharmacological actions of anlotinib has not been fully elucidated, which represents a limitation of the present study. The current *in vitro* model lacks endothelial cells and immune components, thereby failing to recapitulate the complex cellular interactions and structural features of the *in vivo* tumor microenvironment. Further validation using well-characterized *in vivo* models is strongly warranted. This constitutes another major limitation of this study.

**Figure 4:**
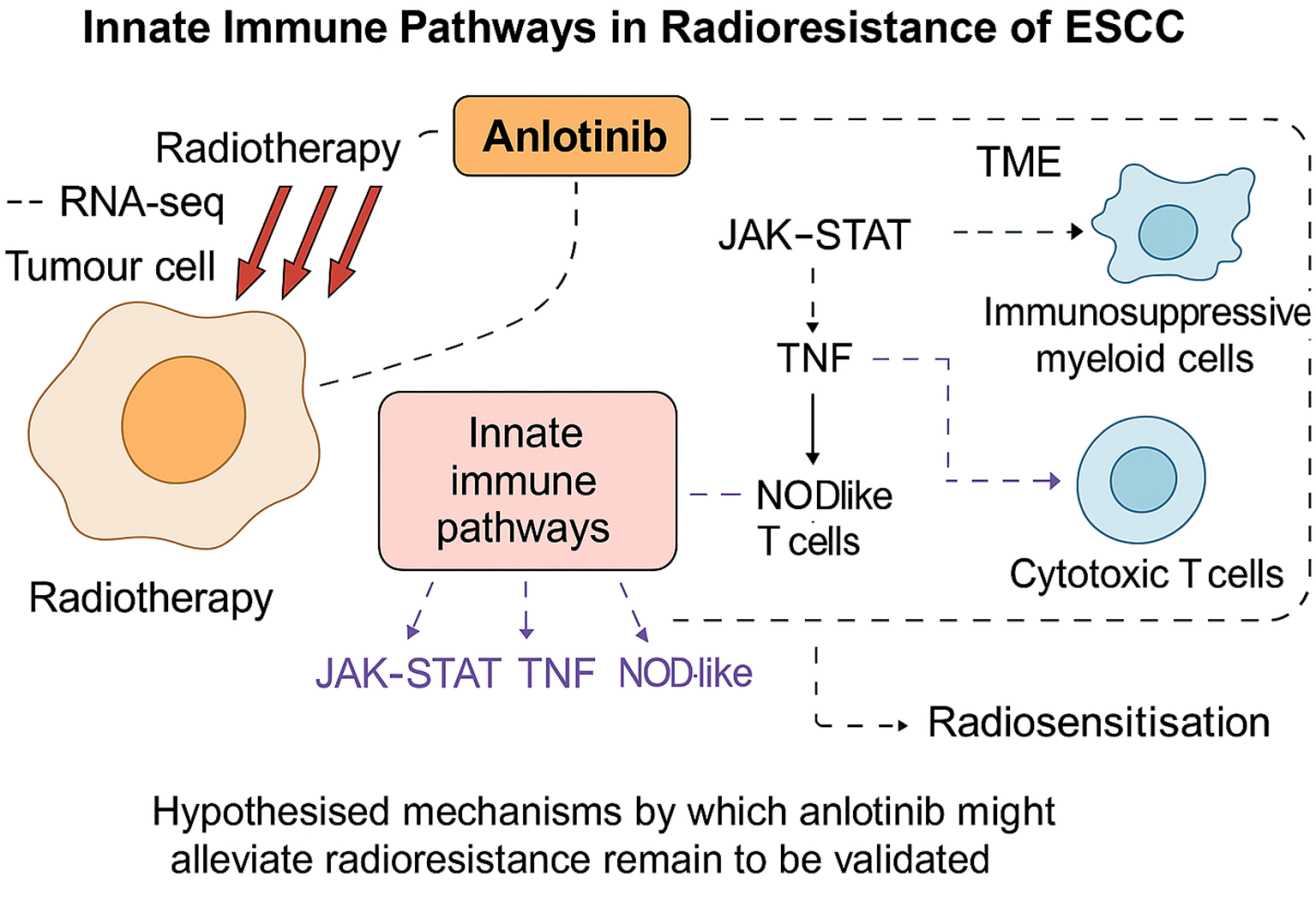
Hypothetical model of anlotinib-enhanced radiosensitisation in ESCC. This schematic summarizes transcriptomic alterations in radioresistant ESCC cells after fractionated irradiation, including activation of innate immune pathways such as the Janus kinase–signal transducer and activator of transcription (JAK-STAT) cascade, nucleotide-binding oligomerization domain (NOD)-like receptor signaling, and cytosolic DNA-sensing pathways. Although anlotinib-specific transcriptomic data were not available, clinical findings and prior studies suggest its potential role in modulating the tumor microenvironment, promoting vascular normalization, and enhancing immune activation. Dashed arrows indicate hypothetical links requiring further validation.

This study has several limitations that need to be acknowledged. First, it is a single-arm, single-center trial with a relatively small sample size, which may lead to selection bias and limit the generalizability of the results. Second, the mechanistic study is only based on *in vitro* cell lines, and no *in vivo* animal experiments or clinical tissue sample verification were performed. Third, no biomarker-guided stratified treatment was set up in the trial, and the predictive biomarkers of anlotinib combined with CRT for ESCC postoperative isolated lymph node recurrence remain to be explored. Fourth, the long-term toxicity data of the regimen are lacking, and further long-term follow-up is needed to evaluate the late adverse events (e.g., radiation pneumonitis, esophageal stricture). Fifth, the study did not explore the combination of anlotinib with immunotherapy, which may be a potential direction to further improve the efficacy. However, the concordance between clinical and transcriptomic data supports the translational relevance of these findings. Future studies should incorporate immunophenotyping, dynamic biomarker monitoring, and stratified treatment approaches to elucidate TME dynamics further and optimize personalized therapy. Multicenter, randomized controlled trials with larger sample sizes are urgently needed to validate the efficacy of this regimen and establish clinical application standards. As acknowledged in the **Figure 4**, direct transcriptomic evidence of anlotinib-induced modulation of JAK-STAT, TNF and cytosolic DNA-sensing pathways remains absent from our *in vitro* cell experiments, and all regulatory links drawn in the hypothetical model are tentative dashed lines requiring prospective validation. Future translational work will collect paired pre- and post-treatment recurrent lymph node tissue specimens to perform multi-omics and single-cell RNA sequencing, which will directly quantify tumor cell, endothelial and immune compartment transcriptomic shifts after anlotinib-combined CRT and functionally verify the immune-radiosensitising pathways proposed in our model.

Although univariate Cox regression showed no significant between-group differences in PFS and OS between weekly paclitaxel plus cisplatin (TP, 79% of patients) and single-agent oral S-1 (21% of patients), we observed divergent treatment tolerance profiles. The TP doublet regimen was associated with higher rates of grade ≥ 3 leukopenia and grade 2–3 gastrointestinal nausea/vomiting, while oral S-1 monotherapy yielded milder myelosuppression and gastrointestinal adverse events, with superior oral treatment adherence and fewer unscheduled supportive care visits. No patients in either group discontinued concurrent CRT due to chemotherapy-related toxicity, confirming both regimens are clinically feasible for salvage concurrent CRT combined with anlotinib in this patient population.

A total of 47 patients were included, among whom 23 developed disease progression during follow-up. Their second-line regimens were individualized according to clinical conditions, mainly including tegafur-based combined immunotherapy, TP chemotherapy and palliative radiotherapy. Post-progression second-line treatments such as immunotherapy and targeted therapy were not collected in this study, which may interfere with OS results. Post-progression treatment was not standardized in our trial and followed routine clinical practice. We acknowledge this limitation, and suggest that subsequent trials should prospectively record relevant treatment information to reduce confounding bias.

In conclusion, this study delivers pioneering combined clinical and preliminary mechanistic data to support the clinical feasibility of anlotinib-combined CRT as a postoperative therapeutic option for patients with nodal recurrent ESCC. Focusing on this biologically high-risk yet surgically accessible disease subset, the concurrent application of anlotinib plus CRT yields encouraging clinical outcomes presumably associated with regulatory effects on angiogenic and immune-related signaling axes. Collectively, these findings lay a preliminary foundation for future biomarker-driven combinatorial treatment exploration in recurrent ESCC, while dedicated preclinical *in vitro* and *in vivo* investigations are still required to systematically validate the underlying molecular mechanisms.

## Conclusions

This prospective phase II study indicates that anlotinib plus CRT constitutes a feasible salvage therapeutic option for patients with postoperative lymph node recurrent ESCC, with encouraging survival endpoints and acceptable adverse events noted in this anatomically defined patient subgroup, which lends preliminary clinical rationale for incorporating antiangiogenic compounds into CRT combinations. Transcriptomic analyses of radioresistant ESCC cell samples identified differential expression changes linked to immune and angiogenic signaling, which may partially explain the observed radiosensitising synergy at the transcriptional level; nevertheless, dedicated *in vitro* and *in vivo* preclinical experiments are still needed to corroborate these correlative molecular findings. Overall, these clinical results merit further confirmation via large-scale randomized controlled trials, while prospective research is required to clarify whether anlotinib-based combinatorial regimens with immune-modulating interventions could reverse radiotherapy resistance among high-risk ESCC patients (43).

## Data Availability

The raw transcriptome sequencing data generated in this study have been deposited in the National Center for Biotechnology Information (NCBI) Sequence Read Archive (SRA) database under BioProject accession number PRJNA1469848.

## Supplementary material

Supplementary Figure 1 Treatment schema of anlotinib-based concurrent CRT.

Supplementary Figure 2 Forest plot comparing PFS and OS according to the lymph node number and maximum diameter.; no statistically significant differences were observed.

Supplementary Figure 3 FPKM distribution, DEG statistics and DEG expression clustering in NC and Ra cells. (A) Density distribution of log_10_(FPKM) values across NC and Ra samples. (B) Counts of DEGs between NC and Ra cells, categorized as total, downregulated, and upregulated. (C) Heatmap of DEGs between NC and Ra cells, with hierarchical clustering based on normalized expression values.

Supplementary Figure 4 GSEA plots showing significantly downregulated pathways in Ra versus NC cells: (A) defense response to virus, (B) positive regulation of immune response, (C) cytokine activity, and (D) receptor signaling pathway via JAK–STAT.

Supplementary Figure 5 **(A)** GO enrichment of DEGs in Ra versus NC cells. **(B)** Heatmap of significant TF influence scores.
