# Supplementary material for: Anlotinib combined with chemoradiotherapy for postoperative isolated lymph node recurrence of ESCC: a phase II study
Source: Front Immunol. 2026 Jul 24;17:1862131. doi: 10.3389/fimmu.2026.1862131 (PMC13447363; doi:10.3389/fimmu.2026.1862131)
Supplement: Supplementary file 1 [file Table1.docx]

Supplementary Table S1. Univariate Cox regression comparing TP versus S-1 chemotherapy

| **Endpoint** | **Comparison** | **HR_CI** | **p_value** |
| --- | --- | --- | --- |
| PFS | TP vs S1 | 2.00 (0.84–4.80) | 0.12 |
| OS | TP vs S1 | 1.36 (0.47–3.90) | 0.57 |
